# Comparative analysis of monocyte-derived dendritic cell phenotype and T cell stimulatory function in patients with acute-on-chronic liver failure with different clinical parameters

**DOI:** 10.3389/fimmu.2023.1290445

**Published:** 2023-12-04

**Authors:** Zhipeng Wu, Hongbo Shi, Lei Zhang, Honglin Shi, Xingzhong Miao, Liangjuan Chen, Yu Chen, Yingmin Ma

**Affiliations:** ^1^ Department of Respiratory and Critical Care Medicine, Beijing Youan Hospital, Capital Medical University, Beijing, China; ^2^ Beijing Institute of Hepatology, Beijing Youan Hospital, Capital Medical University, Beijing, China; ^3^ Beijing Engineering Research Center for Precision Medicine and Transformation of Hepatitis and Liver Cancer, Beijing, China; ^4^ Fourth Department of Liver Disease (Difficult & Complicated Liver Diseases and Artificial Liver Center), Beijing You’an Hospital, Capital Medical University, Beijing, China; ^5^ Department of Nephrology, Shanxi Provincial People's Hospital, The Affiliated People's Hospital of Shanxi Medical University, Shanxi, China; ^6^ Department of Traditional Chinese Medicine, Qinhuangdao Shanhaiguan People's Hospital, Hebei, China

**Keywords:** monocyte-derived dendritic cell, phenotype, T cell stimulatory function, acute-on-chronic liver failure, clinical parameters

## Abstract

**Background:**

Acute-on-Chronic Liver Failure (ACLF) patients experience systemic inflammation as well as immune dysfunction and exhaustion. The phenotype and functionality of monocyte-derived dendritic cells in ACLF patients with different clinical parameters have not been elucidated.

**Methods:**

This study included 37 cases of ACLF, 20 cases of Chronic Hepatitis B (CHB) patients, and 12 healthy controls. Demographic and laboratory parameters were collected from the enrolled patients. Peripheral blood samples were obtained from the participants. Monocyte-derived dendritic cells were induced and cultured, followed by co-culturing with T cells from the patients. Cell surface markers and intracellular markers were analyzed using flow cytometry. The relationship between these markers and clinical parameters was compared.

**Results:**

Our study found that ACLF patients had lower expression levels of HLA-DR, CD86, and CD54 on monocyte-derived dendritic cells compared to both CHB patients and healthy controls. IL-4, GM-CSF, and alcohol were found to promote the expression of HLA-DR, CD86, and CD54 on monocyte-derived dendritic cells. In ACLF patients, higher levels of procalcitonin (PCT), lower levels of albumin, decreased prothrombin activity and deceased patients were associated with lower expression of HLA-DR, CD86, and CD54 on monocyte-derived dendritic cells. Peripheral blood mononuclear cells (PBMCs), after removing adherent cells, were co-cultured with monocyte-derived DC. Our study revealed that patients with infection and low albumin levels exhibited a decreased proportion of T cell subsets within PBMCs. Additionally, these patients’ T cells showed lower levels of Ki-67 and interferon-gamma (IFN-γ) production.

**Conclusion:**

ACLF patients exhibit varying clinical states, with differences in the phenotype and the ability of monocyte-derived dendritic cells to stimulate T cells. Alcohol can stimulate the maturation of monocyte-derived dendritic cells.

## Introduction

Acute-on-chronic liver failure (ACLF) is a severe form of acute decompensated liver cirrhosis, characterized by the failure of one or more major organ systems (liver, kidney, circulation/respiration/brain, and coagulation), as well as systemic inflammation that may be caused by acute precipitating factors (either intrahepatic or extrahepatic stimuli, or both) (1, 2). In Europe, alcohol-related factors are the primary contributors to ACLF, while in Asia, approximately 70% of ACLF cases are caused by Hepatitis B Virus (HBV) (3, 4). Despite advances in antiviral medications and life support technologies, the mortality rate associated with ACLF remains high (5, 6).

Until now, there are still many unknowns regarding the pathophysiology of ACLF (7). Currently, we know that systemic inflammation plays a crucial role in driving ACLF (8, 9). However, our understanding of how systemic inflammation develops and its impact on organ function is limited. Additionally, another observed immune dysfunction in ACLF is immune cell paralysis, which can lead to bacterial infections and worsen the overall condition (10, 11). Evidence of excessive inflammatory response includes elevated levels of C-reactive protein and white blood cells in the plasma, increased levels of inflammatory cytokines (IL-6 and TNF-α) and anti-inflammatory cytokines (IL-10 and IL-1ra) in the circulatory system, as well as activation of macrophages (8). Evidence of immune dysfunction includes impaired neutrophil function in ACLF patients (12), decreased expression of HLA-DR in monocytes (mHLA-DR) (13), low levels of TNF-α production in response to LPS stimulation (14), and recent studies have also found a reduction in Natural Killer (NK) cells and their association with poor prognosis in ACLF patients (6).

Monocyte-derived dendritic cells (MoDCs) play a critical role in the immune system (15, 16). They serve as important antigen-presenting cells, capable of capturing foreign antigens and activating T cells, thereby triggering an immune response. Additionally, MoDCs produce various cytokines and chemokines that regulate the activity and interaction of immune cells (17). Monocytes and DC cells play important roles in the development of ACLF (18). Studies have shown that ACLF patients experience a decrease in HLA-DR expression on peripheral blood monocytes as the disease worsens, and severe cases exhibit reduced secretion of pro-inflammatory cytokines IL-1β and TNF-α (19). Additionally, research suggests that DCs migrate from the blood to the liver in ACLF patients, where they suppress the secretion of IFN-γ by CD8 T cells, thereby reducing liver damage (16). However, the exact role of MoDCs in ACLF development remains unclear, especially regarding the status of MoDCs in ACLF patients with different clinical conditions.

To address this question, we compared the phenotype of MoDCs between patients with acute-on-chronic liver failure (ACLF), chronic hepatitis B (CHB), and healthy controls. We then categorized ACLF patients based on infection, albumin levels, prothrombin activity, and outcome status. We further compared the functional properties of MoDCs and their ability to promote T cell responses in ACLF patients with different clinical profiles.

## Materials and methods

### Study participants

This study enrolled a total of 37 patients with ACLF who were treated at Beijing Youan Hospital, Capital Medical University, from March 2022 to October 2022. Additionally, 20 patients with Chronic Hepatitis B (CHB) and 12 healthy individuals were included as control groups. The ACLF patients were monitored until either their death or discharge from the hospital. The diagnosis of ACLF was based on the criteria established by the Asian Pacific Association for the Study of the Liver, which defines it as an acute liver injury characterized by jaundice (serum bilirubin ≥ 85.5 μmol/L [5 mg/dL]) and impaired coagulation (prothrombin activity < 40% or international normalized ratio ≥ 1.5), accompanied by clinical manifestations of ascites and/or encephalopathy within four weeks in patients with previously diagnosed or undiagnosed chronic liver disease/cirrhosis (3). The diagnosis of CHB followed the guidelines outlined in the Chronic Hepatitis B Prevention and Treatment Guidelines (2019 edition) (4). Individuals with concurrent HIV infection, hepatitis C virus (HCV) or hepatitis G virus (HGV) infections, autoimmune liver diseases, malignancies, age below 18 or above 80 years, and pregnant women were excluded from the study. Due to the significant impacts of immune modulators and steroids on the immune system, the patients included in our study did not receive these medications, or the blood samples were collected prior to the administration of these medications. The study collected clinical characteristics and recorded 28-day mortality data. Demographic information and baseline laboratory parameters, including gender, age, and various laboratory values such as prothrombin time activity, white blood cell count, hemoglobin, calcitonin, alanine aminotransferase, aspartate aminotransferase, platelet count, total bilirubin, direct bilirubin, and albumin, and HBsAg status, HBeAg status, and HBV DNA levels were documented for the enrolled patients (**Table 1**). The development of acute injury in HBV-ACLF and the treatment history of antiviral therapy are presented in **Tables S1**, **S2**, respectively. The study received approval from the Ethics Committee of Beijing Youan Hospital, Capital Medical University, and informed consent was obtained from patients or their family members in cases the patients themselves were unable to sign the consent form before participating in the study.

**Table 1:** Essential characteristics of HC and patients with CHB and ACLF in our study.

Characteristics	ACLF (n=37)			CHB (n=20)	HC (n=12)
	Total (n=37)	Survivors (n=21)	Non-survivors (n=16)		
Demography characters					
Sex, male (N,%)	27 (73.0%)	16 (76.2%)	11 (68.8%)	15 (70.0%)	6 (50%)
Age (years)	55 (45,56)	54 (43,56)	56 (54,60)	57 (45,62)	52 (48,54)
Laboratory parameters					
Prothrombin time activity (%)	33 (27,36)	36 (34,39)	26 (23,32)	72 (62,88)	NA
White blood cell (×109/L)	7 (5,10)	8 (6,12)	6 (4,8)	7 (4,8)	NA
Procalcitonin (ng/mL)	1 (0,1)	1 (0,1)	1 (0,1)	1 (0,1)	NA
Aspartate aminotransaminase (U/L)	179 (109,230)	118 (98,206)	196 (172,249)	76 (55,87)	NA
Alanine aminotransferase (U/L)	113 (55,163)	75 (52,115)	148 (118,206)	45 (34,75)	NA
Total bilirubin (μmol/L)	345 (22)	281 (25)	430 (27)	68 (4)	NA
Direct bilirubin (μmol/L)	198 (13)	162 (16)	245 (17)	36 (3)	NA
Albumin (g/L)	26 (1)	29 (1)	22 (1)	32 (1)	NA
Etiology					
HBsAg-positive n (%)	37 (100.0%)	21 (100.0%)	16 (100.0%)	20 (100.0%)	NA
HBeAg-positive n (%)	20 (54.1%)	11 (52.4%)	9 (56.3%)	11 (55.0%)	NA
HBV DNA (log_10_ IU/mL)	4.67 (4.25,5.33)	4.52 (4.20,5.21)	5.28 (4.38,6.0)	4.28 (3.17,5.13)	NA

The normality of continuous variables was assessed using the Shapiro-Wilk test. Normally distributed continuous variables were displayed as mean ± standard deviation (SD), non-normally distributed continuous variables were displayed as median and interquartile range (IQR). Categorical variables were expressed as counts with percentages. HC, Healthy control; CHB, Chronic Hepatitis B; ACLF, Acute-on-Chronic Liver Failure; NA, not applicable.

### Blood sample processing and cell culture

Under sterile conditions, 10 mL of peripheral whole blood sample was collected using purple-capped anticoagulant tubes. Peripheral blood mononuclear cells (PBMCs) were separated from the blood sample using Ficoll-Paque PLUS (GE Healthcare, Uppsala, Sweden) density gradient centrifugation. In brief, whole blood was mixed with RPMI 1640 medium (Gibco, cat. no.: C11875500CP) and lymphocyte separation solution (STEMCELL, cat. no.: 7851) in a 1:1:1 ratio, transferred to Leucosep centrifuge tubes (Greiner Bio-One, Germany), and centrifuged at 2000 rpm for 10 minutes at 21°C without braking. The white cell pellet containing PBMCs was collected, and the cells were washed twice with 10 mL of fresh RPMI-1640. Cell counting was performed, and the cells were resuspended at a density of 2×10^6 cells/mL in serum-free culture medium and transferred to a 6-well culture plate with 2 mL of serum-free culture medium. The culture plate was then incubated at 37°C for 2 hours. After incubation, monocytes adhered to the culture plate while lymphocytes remained in suspension. The supernatant containing lymphocytes was collected and discarded, and the cell suspension was frozen and stored using RPMI-1640 (10%) + FBS (80%) (Gibco, cat. no.: 10099141) + dimethyl sulfoxide (DMSO; 10%) (Sigma, USA, cat. no.: D2650) as the freezing medium. The cells were divided into small aliquots and stored at -80°C for future use.

The immature MoDCs from monocytes were generated using a previously established method (20). Monocytes were cultured in 6-well cell culture plates using VIVO medium (Lonza, cat. no.: 04-418Q) for 6 days. The medium was supplemented with 10% fetal bovine serum (FBS), IL-4 (Peprotech, cat. no.: AF-200-04-20UG) at a concentration of 50 ng/mL, and GM-CSF (Peprotech, cat. no.: AF-300-03-20UG). After washing the immature dendritic cells, they were further cultured in RPMI-1640 medium supplemented with 10% FBS and 1% penicillin-streptomycin-glutamine solution (PSG) in a 96-well plate. On the seventh day, the cells were stimulated again using a combination of IL-4 and GM-CSF or cytokines with alcohol (30 μl/ml). On the eighth day, the cells were collected and washed twice with PBS. Cell aggregates were removed using a cell filter. The dendritic cells were co-cultured with lymphocytes at a ratio of 1:10, and autologous lymphocyte suspension and IL-2 (Peprotech, cat. no.: AF-200-02) at a concentration of 50 U/L were added. The co-culture was maintained for a total of 4 days.

### Flow cytometry

For cell surface and intracellular staining, standard protocols were followed using monoclonal antibodies. Cells and antibodies were co-incubated on ice in the dark for 30 minutes. For intracellular staining of cytokines, cells were permeabilized using the Cytofix/Cytoperm Kit (BD Biosciences, San Jose, CA, USA) for 30 minutes on ice, followed by an additional 30-minute incubation with antibodies in the dark at 4°C. The following antibodies were used: Anti-HLA-DR-APC (BD, cat. no.: 339194), anti-human CD54-APC (BD, cat. no.: 561899), anti-human CD86-FITC (BD, cat. no.: 560958), anti-human CD3-Percp (BD, cat. no.: 552851), anti-Human CD4-PE (BD, cat. no.: 557344), anti-Human CD8-FITC (BD, cat. no.: 555366), anti-human Ki-67-APC (BioLegend, cat. no.: 350514), and anti-IFN-gamma-APC (BD, cat. no.: IC285A-100). Imaging flow cytometry was performed using the ImageStreamX MarkII quantitative imaging flow cytometer, while conventional flow cytometry was performed using the BD FACS Calibur flow cytometer.

### Statistical analyses

Data analysis and graphing were performed using GraphPad Prism (Version 9.0, La Jolla, CA, USA). The data were presented as mean with standard deviation (SD) and analyzed using two-way analysis of variance (ANOVA). Significance levels were denoted as *P < 0.05, **P < 0.01, ***P < 0.001, and ****P < 0.0001.

## Result

### The proportion of mature moDCs in patients with acute-on-chronic liver failure is lower compared to those with chronic hepatitis B and healthy controls

**Figure 1:**
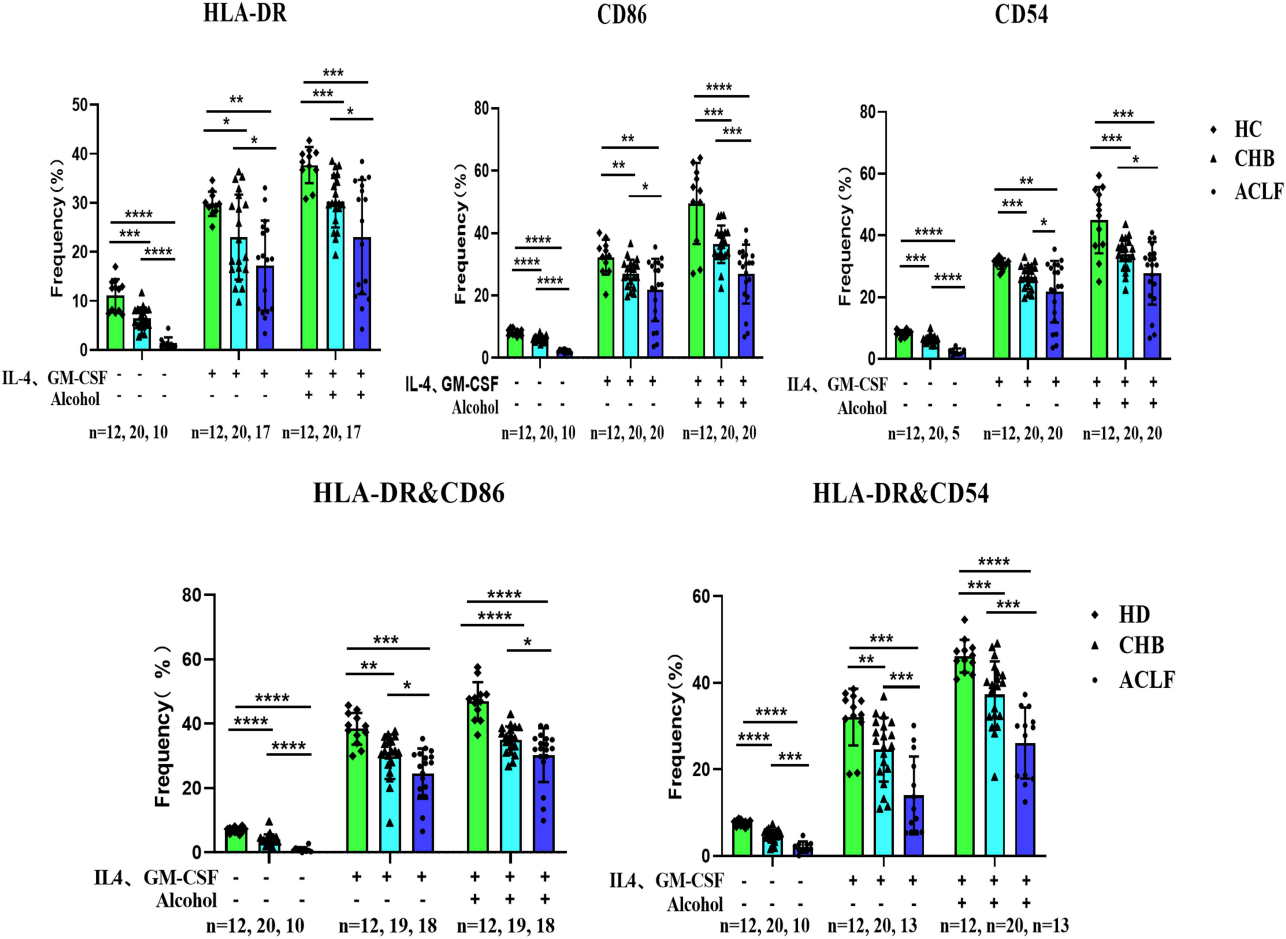
Summary data of HLA-DR, CD86, CD54 expression on moDCs from HC, CHB and ACLF with or without stimulation. moDCs: Monocyte-derived dendritic cells; HC: Healthy control; CHB: Chronic Hepatitis B; ACLF: Acute-on-Chronic Liver Failure; HLA-DR: human leukocyte antigen-DR; GM-CSF: Granulocyte-Macrophage Colony-Stimulating Factor; SD: Standard Deviation. Data analyzed by two-way analysis of variance (ANOVA), displayed as Mean with SD. *P <0.05, **P <0.01, ***P <0.001 and ****P <0.0001.

In order to investigate the changes in monocyte-derived dendritic cells in patients with ACLF, we compared the monocyte-derived dendritic cells from peripheral blood among three groups: healthy controls, CHB patients, and ACLF patients. A total of 37 ACLF cases, 20 CHB cases, and 12 healthy individuals were included in the study, and the necessary characteristics are presented in **Table 1**. Human leukocyte antigen-DR (HLA-DR), along with CD86 and CD54, are important surface markers for dendritic cell maturation. Our study found that the expression of HLA-DR, CD86, and CD54 in ACLF patients was lower compared to both CHB patients and healthy controls. IL-4 and GM-CSF can stimulate dendritic cell maturation, and our previous research has shown that alcohol can also induce DC maturation (**Figures S1**, **S2**). After adding these stimulatory factors, the expression of HLA-DR, CD86, and CD54 on DCs increased, but the ACLF group still exhibited lower expression compared to the CHB and HC groups (**Figure 1** and **Figure S3**).

### The proportion of mature moDCs in patients with acute-on-chronic liver failure varies based on different clinical parameters

**Figure 2:**
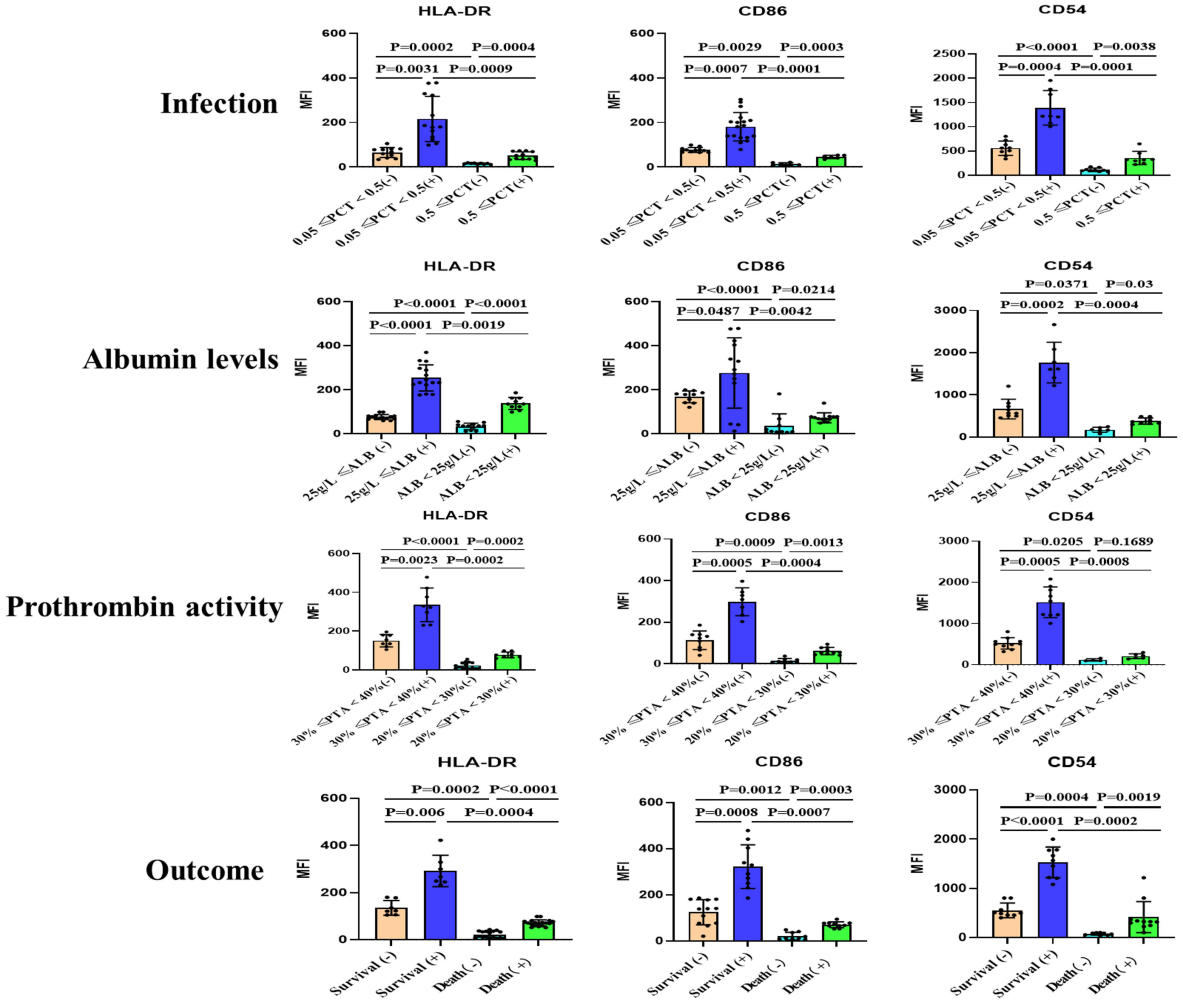
Summary data on the surface expression of HLA-DR, CD86, and CD54 on monocyte-derived dendritic cells (moDCs) from patients with acute-on-chronic liver failure (ACLF) with or without stimulation. Grouping was performed based on infection, Albumin levels, Prothrombin activity and Outcome. (+): Stimulated with alcohol. Representative data was determined as mean fluorescence intensity (MFI). moDCs: Monocyte-derived dendritic cells; ACLF: Acute-on-Chronic Liver Failure; HLA-DR: human leukocyte antigen-DR; PCT: Procalcitonin; SD: Standard Deviation. Data analyzed by two-way analysis of variance (ANOVA), displayed as Mean with SD.

In order to compare the functional differences of moDCs in ACLF patients based on different clinical parameters, we grouped the patients according to their levels of procalcitonin (PCT), albumin, Prothrombin activity (PTA), and whether they had deceased. The study revealed a correlation between the maturity of moDCs and these clinical indicators. Patients with high PCT levels, low albumin levels, low coagulation factor activity, and deceased patients exhibited lower expression of HLA-DR, CD86, and CD54 on moDCs. We also observed that alcohol can stimulate the maturation of moDCs. However, even after alcohol stimulation, patients with high PCT levels, low albumin levels, low PTA, and deceased patients still showed lower expression of HLA-DR, CD86, and CD54 (**Figure 2** and **Figures S4-S7**).

### The subsets of T cells, their proliferation, and interferon-γ production after co-culture with monocyte-derived dendritic cells are associated with the grouping based on clinical parameters

**Figure 3:**
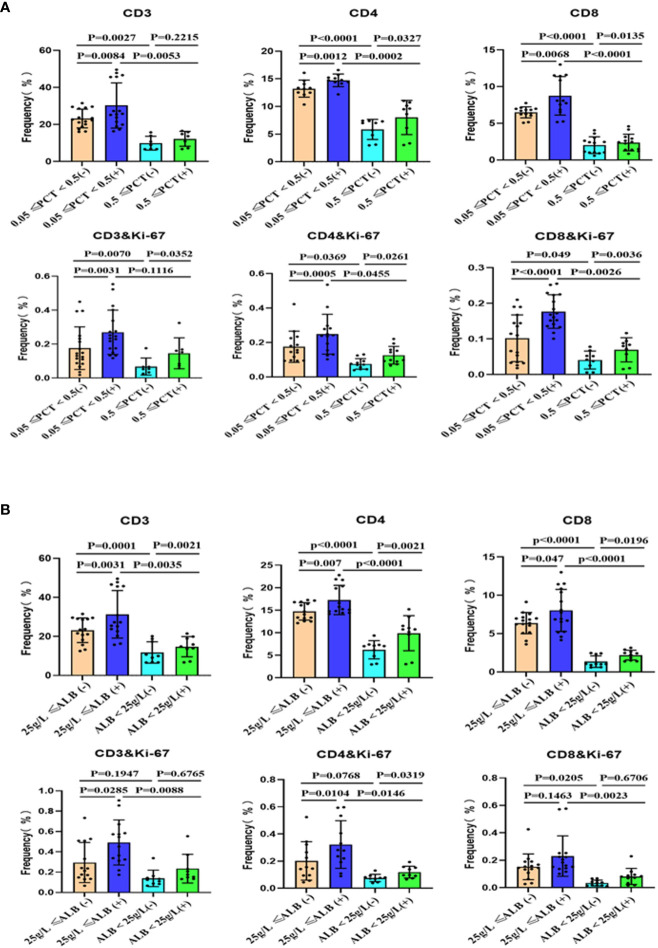
Subsets of peripheral blood T cells and their proliferation after co-culture with moDCs from ACLF with or without stimulation. Summary of the data for the groups based on infection **(A)** and albumin levels **(B)**. (+): Stimulated with alcohol. moDCs: Monocyte-derived dendritic cells; ACLF: Acute-on-Chronic Liver Failure; T: T lymphocytes; PCT: Procalcitonin; SD: Standard Deviation. Data analyzed by two-way analysis of variance (ANOVA), displayed as Mean with SD.

**Figure 4:**
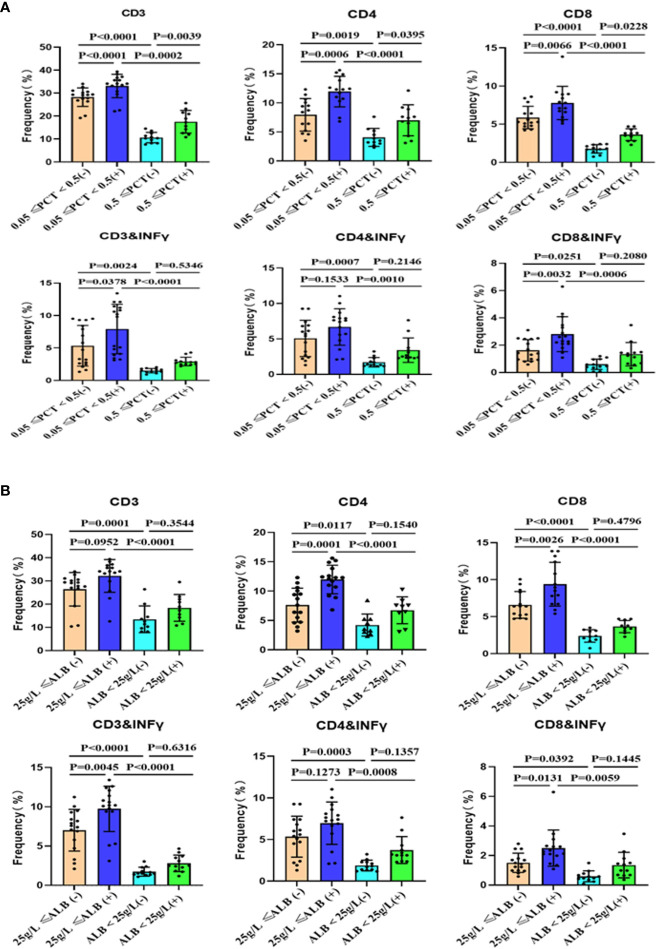
Subsets of peripheral blood T cells and their interferon-γ production after co-culture with mo-DCs from ACLF with or without stimulation. Summary of the data for the groups based on infection **(A)** and albumin levels **(B)**. (+): Stimulated with alcohol. mo-DCs: Monocyte-derived dendritic cells; ACLF: Acute-on-Chronic Liver Failure; T: T lymphocytes; PCT: Procalcitonin; SD: Standard Deviation. Data analyzed by two-way analysis of variance (ANOVA), displayed as Mean with SD.

To compare the stimulatory capacity of monocyte-derived dendritic cells (moDCs) on T cells among patients with different clinical parameters, we grouped the patients based on their levels of procalcitonin (PCT) and albumin. Peripheral blood monocytes, after removing adherent cells, were co-cultured with monocyte-derived DC. Our study found that patients with infection and low albumin levels exhibited a decreased proportion of T cell subsets within PBMCs. Additionally, these patients’ T cells showed lower levels of Ki-67 (**Figure 3** and **Figure S8**) and interferon-γ production (**Figure 4** and **Figure S9**). Alcohol stimulation can enhance the stimulatory effect of monocyte-derived DC on T cells, thereby increasing T cell proliferation and interferon-γ production. However, compared to the non-infection group and non-low albumin group, the infection group and low albumin group still demonstrated lower levels of Ki-67 and interferon-γ production in T cells.

## Discussion

Dendritic cells (DCs) are an important cell type in the immune system, responsible for antigen presentation and promoting immune responses by activating specific T cells (15). Dendritic cells possess highly differentiated cell surface molecules, including HLA-DR, CD86, and CD54, which play important roles in the process of dendritic cell activation of T cells (21–25). This study found that patients with ACLF exhibited significantly reduced expression of surface markers HLA-DR, CD86, and CD54 compared to patients with CHB or healthy individuals. We categorized the patients based on their infection status, albumin levels, prothrombin activity, and outcomes, and found that ACLF patients with infections, low albumin levels, low prothrombin activity, and poor prognosis had significantly decreased surface marker expression on monocyte-derived dendritic cells. When co-cultured with autologous T cells, dendritic cells from these patients showed a significant impairment in their ability to stimulate T cell proliferation and INF-γ production. Additionally, our research revealed that alcohol can effectively promote the maturation of monocyte-derived dendritic cells, thereby enhancing T cell proliferation and INF-γ production.

Immunological dysfunction and exhaustion are important characteristics of ACLF (18). Research has indicated that monocytes in ACLF patients undergo exhaustion due to bioenergetic failure (26). Furthermore, research has demonstrated that monocytes and dendritic cells are impaired in severe alcoholic hepatitis. This immune dysfunction is associated with a higher risk of infection and mortality (27). Another study has shown a correlation between impaired TLR3 signaling in MoDCs and disease severity in ACLF patients. In addition, there was a significant correlation observed between the expression of TLR3 signaling components (TLR3/IFN-β) in individual ACLF patients and markers of disease severity (total bilirubin and prothrombin activity) (28). However, there is still a lack of research on the clinical characteristics of ACLF patients and the features of monocyte-derived dendritic cells.

Prothrombin activity is an important measure of disease severity in ACLF patients and is included in the diagnostic criteria for ACLF. It is also associated with patient prognosis (1, 3). Infection may serve as a triggering factor for ACLF and can contribute to poor patient outcomes (29). Procalcitonin is a significant diagnostic marker for bacterial infections in clinical practice (30, 31). Albumin is commonly used to assess the nutritional status of patients (32, 33). Our study found that these clinical parameters, including mortality, are associated with the functional status of monocyte-derived dendritic cells in ACLF patients.

For ACLF patients, infection can be a contributing factor to disease progression and is commonly associated with complications and poor prognosis (11, 29, 34, 35). Studies have indicated that patients with chronic hepatitis C virus (HCV) infection exhibit decreased levels of DC cells in peripheral blood and reduced release of IFN-alpha compared to healthy individuals (36). The diminished function of DCs may promote susceptibility to infection, and severe infections can lead to excessive activation of immune function, resulting in immune exhaustion and further disease aggravation (18).

Furthermore, research has shown that during the progression of ACLF, the immune response shifts from an initial severe pro-inflammatory phase to an anti-inflammatory phase. The pro-inflammatory cytokines IL-1β and TNF-α secreted by monocytes are increased in the early stage and decreased in the later stage (19). In summary, the interaction between infection and reduced DC function is likely a mutually reinforcing process.

The level of albumin is an important indicator of the clinical nutritional status of patients (1). Currently, specific data on ACLF is lacking, but it can be inferred that malnutrition and muscle wasting are common in most ACLF patients. Studies focused on decompensated cirrhosis patients have shown a high prevalence of malnutrition and muscle wasting, ranging from 22% to 62% reported incidence (37). Extensive data has demonstrated that malnutrition is an independent risk factor for bacterial infection, further decompensation, and mortality in decompensated cirrhosis patients (38, 39). Therefore, special attention needs to be given to the nutritional status of ACLF patients (40). However, there is a lack of research on the relationship between nutritional status and immune status in these patients. Our study, for the first time, suggests a potential association between albumin levels and the maturation of monocyte-derived dendritic cells in patients, and the underlying mechanisms require further investigation.

Prothrombin activity is one of the indicators used to assess clotting function (1). In patients with acute-on-chronic liver failure, there is a correlation between prothrombin activity, infection, and poor prognosis (41–43). While prothrombin activity is primarily used to assess clotting function, there is extensive interaction between the clotting system and the immune system (44). A study on HBV-ACLF revealed a correlation between white blood cell count, lymphocytes, and peripheral T lymphocytes with prothrombin activity (PTA) (45). Another study indicated a negative correlation between plasma sCD163 levels and prothrombin activity (46). There exists a complex interrelationship between prothrombin activity and immune function. Further research is needed to delve into the interactions between the clotting system and the immune system in order to better understand their roles in disease development and treatment.

Research on the impact of alcohol on dendritic cell (DC) function has been conducted. It has been found that chronic alcohol-induced liver disease can inhibit DC function (47). Additionally, studies have indicated that alcohol can directly interfere with DC function, suppressing their ability to combat pathogens. This may result in a weakened immune system and increased susceptibility to infections (48). Both *in vivo* and *in vitro* studies have explored the effects of ethanol on DC function, revealing its influence on the generation, expression of co-stimulatory molecules, and functionality of both plasmacytoid and myeloid dendritic cell subsets (49). However, our research indicates that alcohol can stimulate the maturation of monocyte-derived dendritic cells. We speculate that the reasons for contradictory research outcomes may be related to factors such as cell sources and the timing of stimulation. Prolonged stimulation, which can lead to functional exhaustion of dendritic cells and subsequent functional suppression, may explain the opposite findings. Our study involved a short-term stimulation. The underlying mechanisms require further investigation.

In summary, this study suggests that even among ACLF patients, who have varying degrees of disease severity, the immune cell function, particularly that of monocyte-derived dendritic cells, may differ. Therefore, it may be necessary to consider different treatment approaches for different patient subtypes.

Our study also has several limitations. Firstly, the sample size was relatively small, and further subgrouping of ACLF patients based on the etiology of their disease was not performed. Secondly, detailed information regarding the clinical treatment of patients was not recorded. Thirdly, we did not extract monocytes specifically from PBMCs of patients and then induce and culture them. Fourthly, further subtyping of dendritic cells was not performed. Fifthly, the specific mechanisms by which alcohol stimulates dendritic cells were not investigated, and this is an area for future research.

## Conclusion

ACLF patients exhibit varying clinical states, with differences in the phenotype and the ability of monocyte-derived dendritic cells to stimulate T cells. Alcohol can stimulate the maturation of monocyte-derived dendritic cells.

## Data availability statement

The original contributions presented in the study are included in the article/**Supplementary Material**. Further inquiries can be directed to the corresponding authors.

## Ethics statement

The studies involving humans were approved by The Ethical Committee of Beijing Youan Hospital. The studies were conducted in accordance with the local legislation and institutional requirements. The participants provided their written informed consent to participate in this study.

## Author contributions

ZW: Conceptualization, Data curation, Formal Analysis, Investigation, Supervision, Writing - original draft, Writing - review & editing. HongbS: Conceptualization, Supervision, Validation, Writing - original draft, Writing - review & editing, Funding acquisition. LZ: Data curation, Formal Analysis, Supervision, Writing - original draft. HonglS: Data curation, Supervision, Writing - original draft. XM: Data curation, Formal Analysis, Supervision, Writing - original draft. LC: Data curation, Formal Analysis, Supervision, Writing - original draft. YC: Conceptualization, Supervision, Writing - review & editing. YM: Conceptualization, Supervision, Writing - review & editing, Funding acquisition.

## Supplementary material

Supplementary Table 1 Acute insults in patients with HBV-ACLF.

Supplementary Table 2 History of antiviral treatment in patients with HBV-ACLF.

Supplementary Figure 1 Imaging cytometry of moDCs from HC, CHB, ACLF with or without stimulation.

Supplementary Figure 2 Imaging cytometry of mo-DCs without stimulation or with alcohol stimulation.

Supplementary Figure 3 Representative dot plots of HLA-DR, CD86, CD54 expression on mo-DCs from HC, CHB and ACLF with or without stimulation.

Supplementary Figure 4–7 Representative data depicting the surface expression of HLA-DR, CD86, and CD54 on monocyte-derived dendritic cells (moDCs) from patients with acute-on-chronic liver failure (ACLF), with or without stimulation. Grouping was performed based on infection (**Figure S4**), albumin levels (**Figure S5**), prothrombin activity (**Figure S6**), and outcome (**Figure S7**).

Supplementary Figure 8 Subsets of peripheral blood T cells and their proliferation after co-culture with moDCs from ACLF with or without stimulation.

Supplementary Figure 9 Subsets of peripheral blood T cells and their interferon-γ production after co-culture with mo-DCs from ACLF with or without stimulation.
