# Supplementary material for: Comparative analysis of monocyte-derived dendritic cell phenotype and T cell stimulatory function in patients with acute-on-chronic liver failure with different clinical parameters
Source: Front Immunol. 2023 Dec 4;14:1290445. doi: 10.3389/fimmu.2023.1290445 (PMC10725902; doi:10.3389/fimmu.2023.1290445)
Supplement: Supplementary Table 1 — Acute insults in patients with HBV-ACLF. [file DataSheet_1.docx]

**Additional file 1**

**Table s1. Acute insults in patients with HBV-ACLF.**

| Acute insults | Antiviral treatment (n=20) | | Naive treatment | Total |
| --- | --- | --- | --- | --- |
|  | sustaining (n = 8) | Drug withdrawal (n=12) | (n=17) | (n=37) |
| Viral breakthrough  /rebound (n) | 8 | 12 | 15 | 35 |
| Infections | 2 | 5 | 9 | 16 |
| Alcohol intake | 1 | 4 | 3 | 8 |
| others (n) | 0 | 2 | 2 | 4 |

Among the 20 patients receiving sustained antiviral treatment (n=8) or undergoing drug withdrawal (n=12), viral breakthrough or rebound occurred. Among the 17 treatment-naïve patients, 15 patients with ACLF experienced viral breakthrough/rebound. In addition to viral breakthrough/rebound, some HBV-ACLF patients may have experienced overlapping acute insults, such as bacterial and/or fungal infections, alcohol intake, or other reasons, as shown in the table.

**Table s2. History of antiviral treatment in patients with HBV-ACLF.**

| Antiviral  treatment | Patients numbers | NAs | Duration of antiviral  treatment(years) | Withdrawl of antiviral  treatment(years) | HBeAg/ HBsAg | Log_10_ HBV  DNA (IU/mL) | Re-start  Antiviral  treatment |
| --- | --- | --- | --- | --- | --- | --- | --- |
| sustaining (n = 8) | P1 | ETV | 8 | / | +/+ | 6.56 |  |
|  | P2 | ETV | 9 | / | +/+ | 4.67 |  |
|  | P3 | ETV | 6 | / | -/+ | 6.38 |  |
|  | P4 | ETV | 3.5 | / | +/+ | 3.21 |  |
|  | P5 | ETV | 8 | / | +/+ | 5.32 |  |
|  | P6 | ETV | 7 | / | -/+ | 4.52 |  |
|  | P7 | ETV | 5.5 | / | -/+ | 5.98 |  |
|  | P8 | ETV | 4 | / | +/+ | 6.02 |  |
| Drug withdrawal (n=12) | P9 | 3TC | 0.5 | 2 | -/+ | 4.34 | ETV |
|  | P10 | 3TC | 1 | >5 | +/+ | 3.34 | ETV |
|  | P11 | AD | 1.5 | 2 | +/+ | 4.21 | ETV |
|  | P12 | AD | >3 | >5 | -/+ | 4.21 | ETV |
|  | P13 | ETV | 1.5 | 2 | -/+ | 5.21 | ETV |
|  | P14 | ETV | >3 | 3 | +/+ | 6.23 | ETV |
|  | P15 | ETV | 2 | 3 | -/+ | 5.32 | ETV |
|  | P16 | ETV | 2 | 2 | +/+ | 5.27 | ETV |
|  | P17 | ETV | >3 | 3 | +/+ | 6.14 | ETV |
|  | P18 | ETV | 0.5 | >5 | -/+ | 3.21 | ETV |
|  | P19 | ETV | 1.5 | >5 | +/+ | 5.28 | ETV |
|  | P20 | ETV | 0.5 | >5 | -/+ | 4.32 | ETV |

Among the patients receiving sustained antiviral treatment (n=8), three patients showed drug resistance, while five patients had poor adherence to the prescribed drugs. Among the patients who had suspended antiviral treatment, twelve patients restarted entecavir (ETV) at a dose of 0.5 mg/day upon diagnosis of ACLF. Nucleoside/nucleotide analogues (NAs) used in the treatment included entecavir (ETV) at a dose of 0.5 mg/day, lamivudine (3TC) at a dose of 100 mg/day, adefovir dipivoxil (AD) at a dose of 10 mg/day, and tenofovir disoproxil fumarate (TDF) at a dose of 300 mg/day.


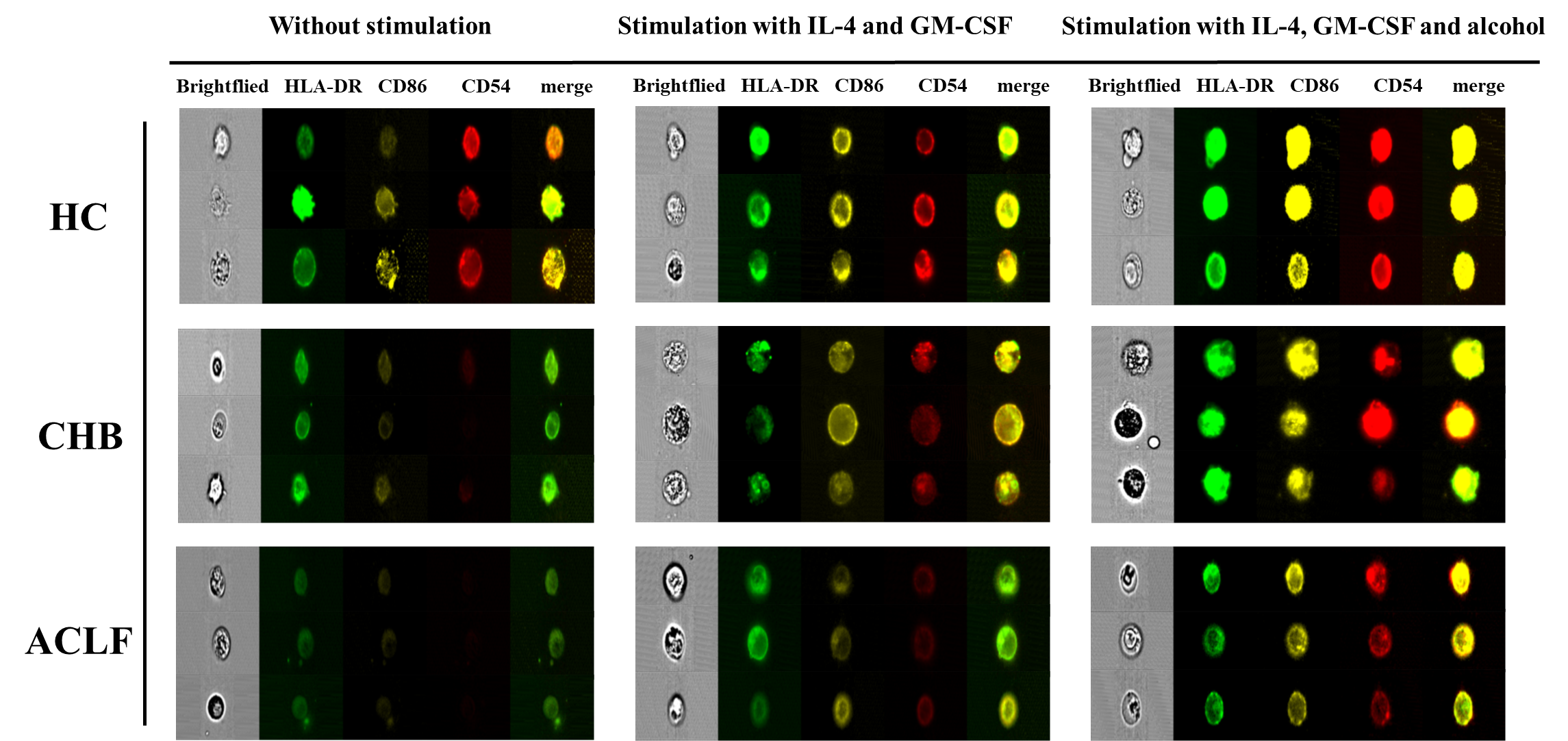


**Figure s1. Imaging cytometry of moDCs from HC, CHB, ACLF with or without stimulation.** (scale bar, 10 μm). Abbreviations: moDCs: Monocyte-derived dendritic cells; HC: Healthy control; CHB: Chronic Hepatitis B; ACLF: Acute-on-Chronic Liver Failure.


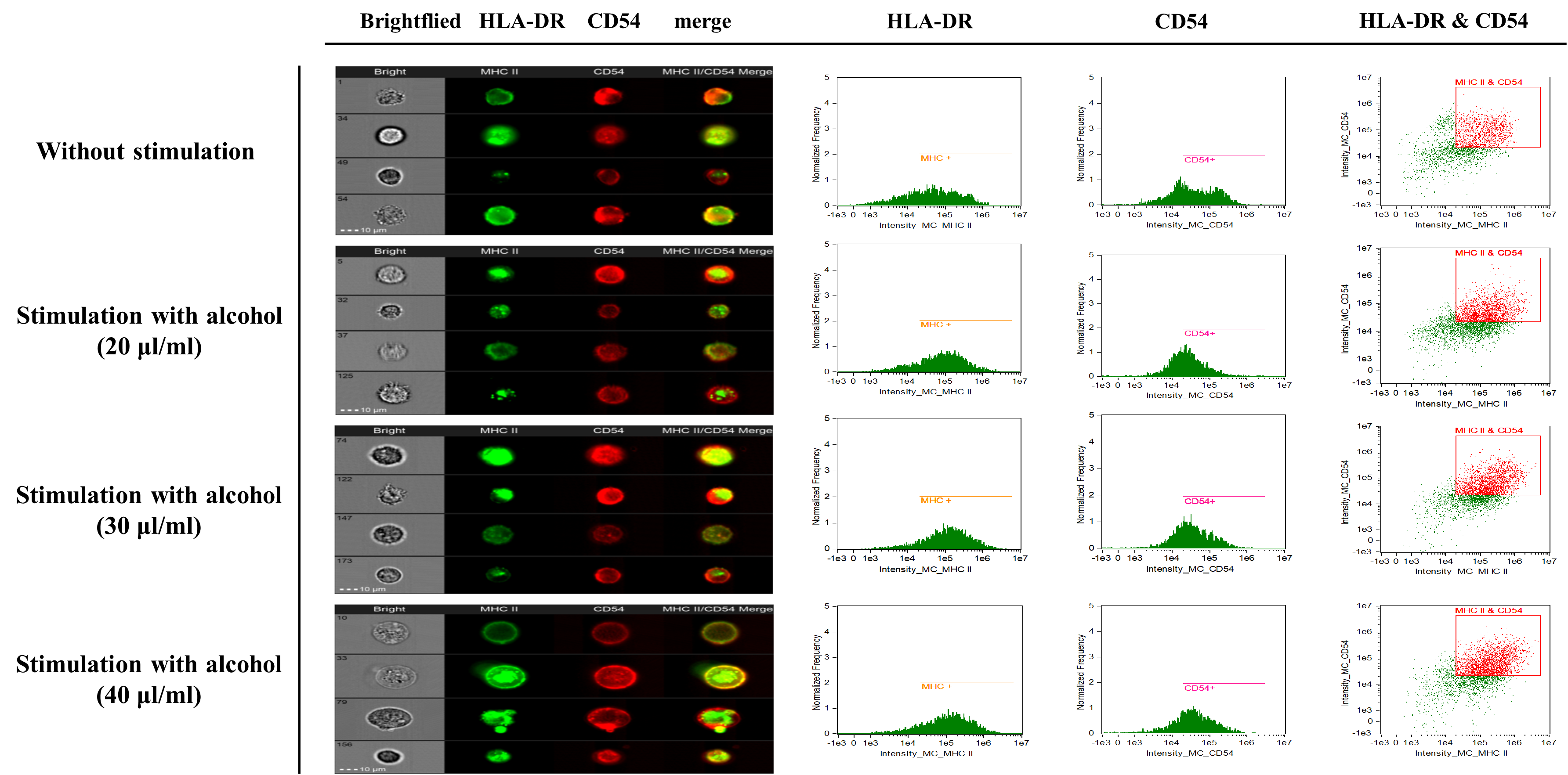


**Figure s2. Imaging cytometry of mo-DCs without stimulation or with alcohol stimulation.** Abbreviations: mo-DCs: Monocyte-derived dendritic cells.


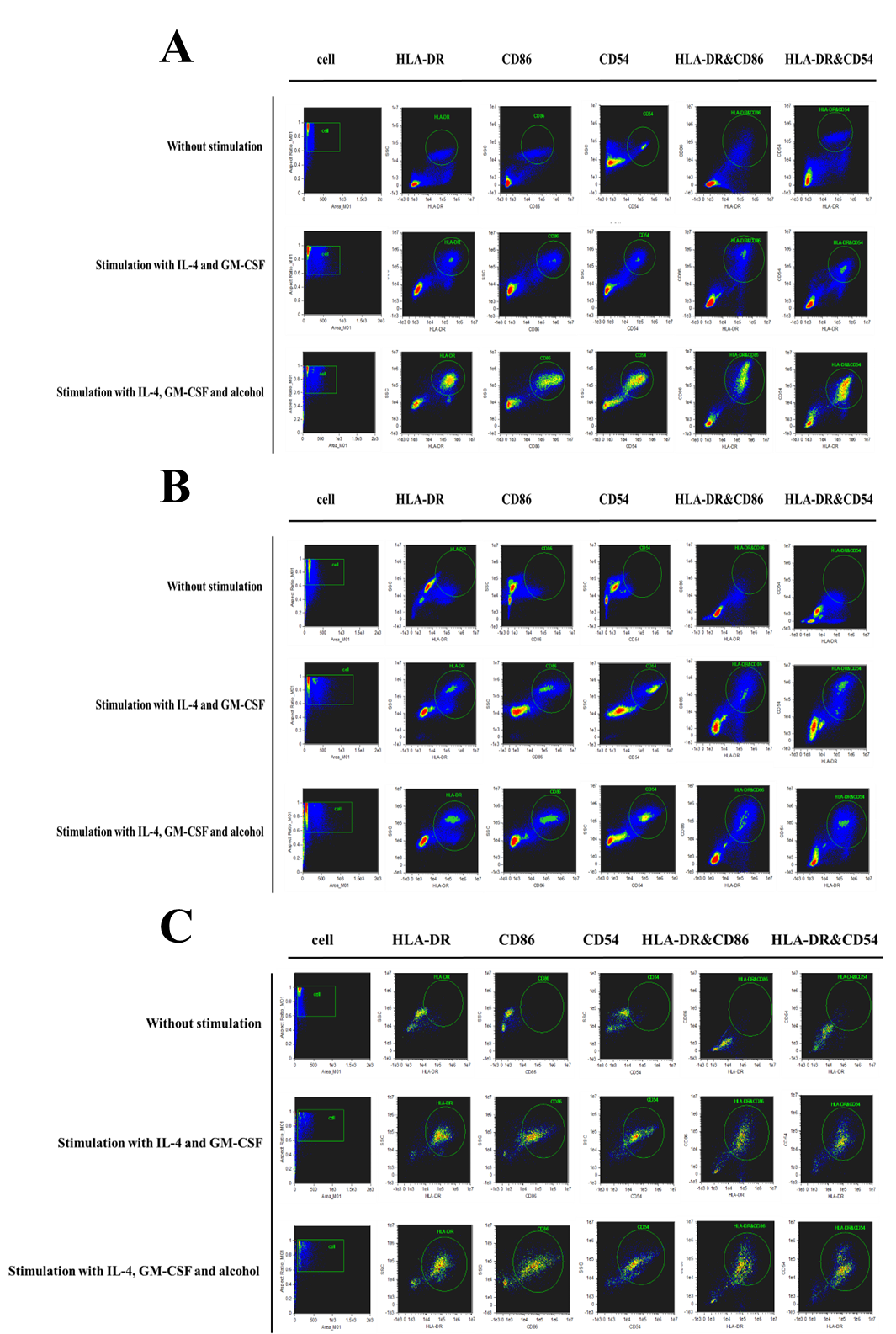


**Figure s3. Representative dot plots of HLA-DR, CD86, CD54 expression on mo-DCs from HC, CHB and ACLF with or without stimulation.** Representative dot plots of mo-DCs for HC (A), CHB (B) and ACLF (C). Abbreviations: mo-DCs: Monocyte-derived dendritic cells; HC: Healthy control; CHB: Chronic Hepatitis B; ACLF: Acute-on-Chronic Liver Failure; HLA-DR: human leukocyte antigen-DR; GM-CSF: Granulocyte-Macrophage Colony-Stimulating Factor; SD: Standard Deviation. Data analyzed by two-way analysis of variance (ANOVA), displayed as Mean with SD. *P <0.05, **P <0.01, ***P <0.001 and ****P <0.0001.


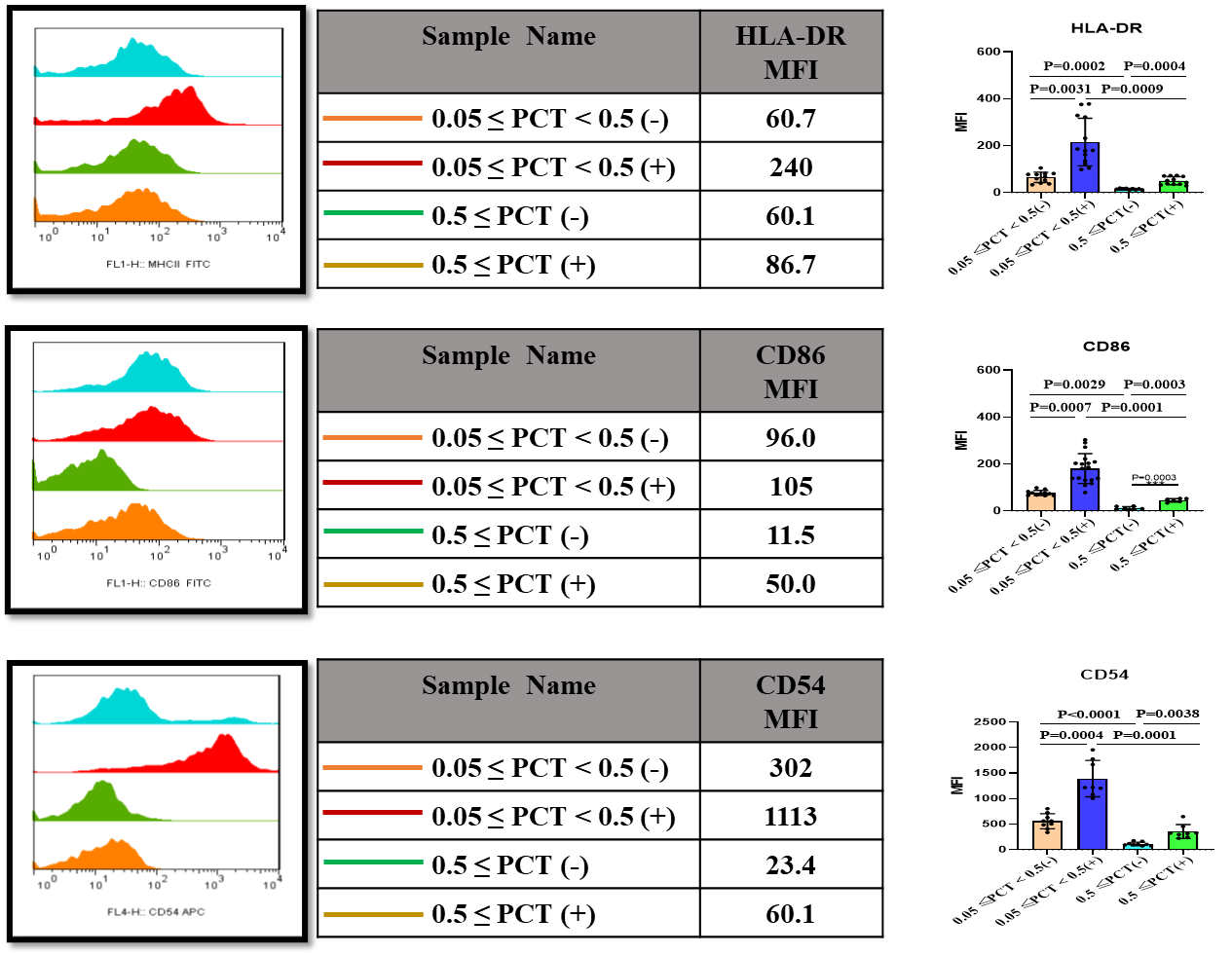


**Figure s4. Representative data depicting the surface expression of HLA-DR, CD86, and CD54 on monocyte-derived dendritic cells (moDCs) from patients with acute-on-chronic liver failure (ACLF), with or without stimulation.** Grouping was performed based on infection. (+): Stimulated with alcohol. Representative data was determined as mean fluorescence intensity (MFI). Abbreviations: moDCs: Monocyte-derived dendritic cells; ACLF: Acute-on-Chronic Liver Failure; HLA-DR: human leukocyte antigen-DR; PCT: Procalcitonin; SD: Standard Deviation. Data analyzed by two-way analysis of variance (ANOVA), displayed as Mean with SD. *P <0.05, **P <0.01, ***P <0.001 and ****P <0.0001.


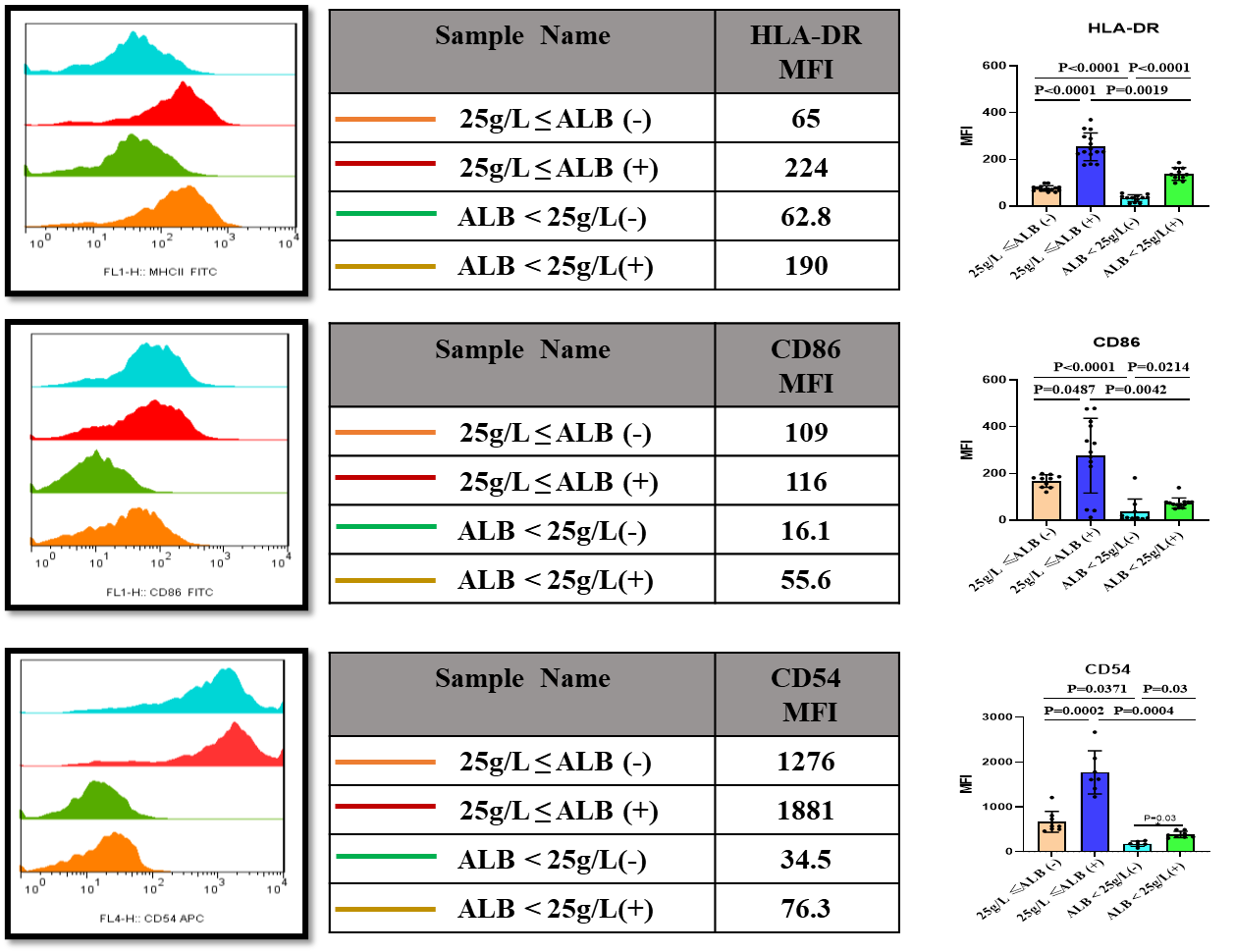


**Figure s5. Representative data depicting the surface expression of HLA-DR, CD86, and CD54 on monocyte-derived dendritic cells (moDCs) from patients with acute-on-chronic liver failure (ACLF), with or without stimulation.** Grouping was performed based on Albumin levels. (+): Stimulated with alcohol. Representative data was determined as mean fluorescence intensity (MFI). Abbreviations: moDCs: Monocyte-derived dendritic cells; ACLF: Acute-on-Chronic Liver Failure; HLA-DR: human leukocyte antigen-DR; PCT: Procalcitonin; SD: Standard Deviation . Data analyzed by two-way analysis of variance (ANOVA), displayed as Mean with SD. *P <0.05, **P <0.01, ***P <0.001 and ****P <0.0001.


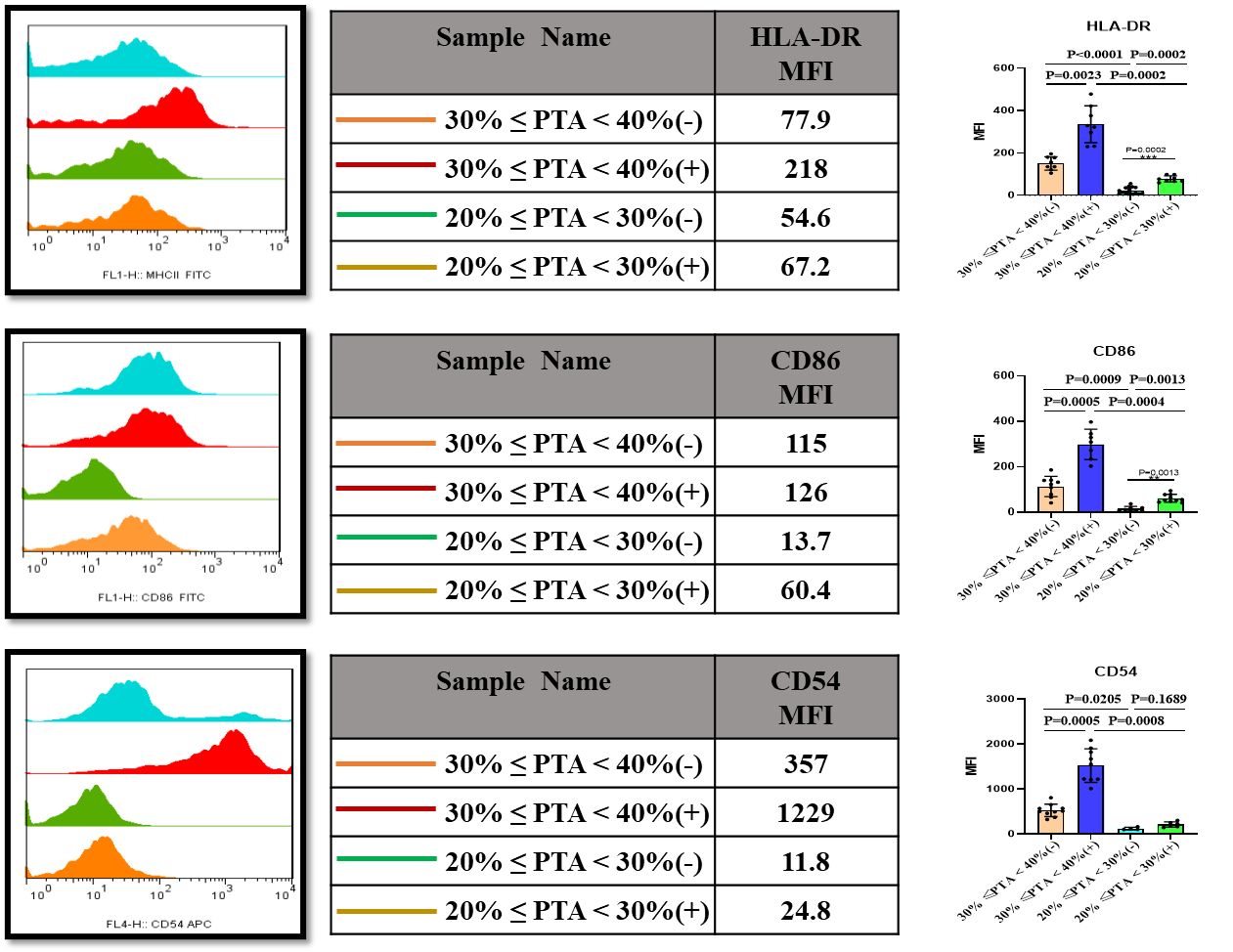


**Figure s6. Representative data depicting the surface expression of HLA-DR, CD86, and CD54 on monocyte-derived dendritic cells (moDCs) from patients with acute-on-chronic liver failure (ACLF), with or without stimulation.** Grouping was performed based on Prothrombin activity. (+): Stimulated with alcohol. Representative data was determined as mean fluorescence intensity (MFI). Abbreviations: moDCs: Monocyte-derived dendritic cells; ACLF: Acute-on-Chronic Liver Failure; HLA-DR: human leukocyte antigen-DR; PCT: Procalcitonin; SD: Standard Deviation . Data analyzed by two-way analysis of variance (ANOVA), displayed as Mean with SD. *P <0.05, **P <0.01, ***P <0.001 and ****P <0.0001.


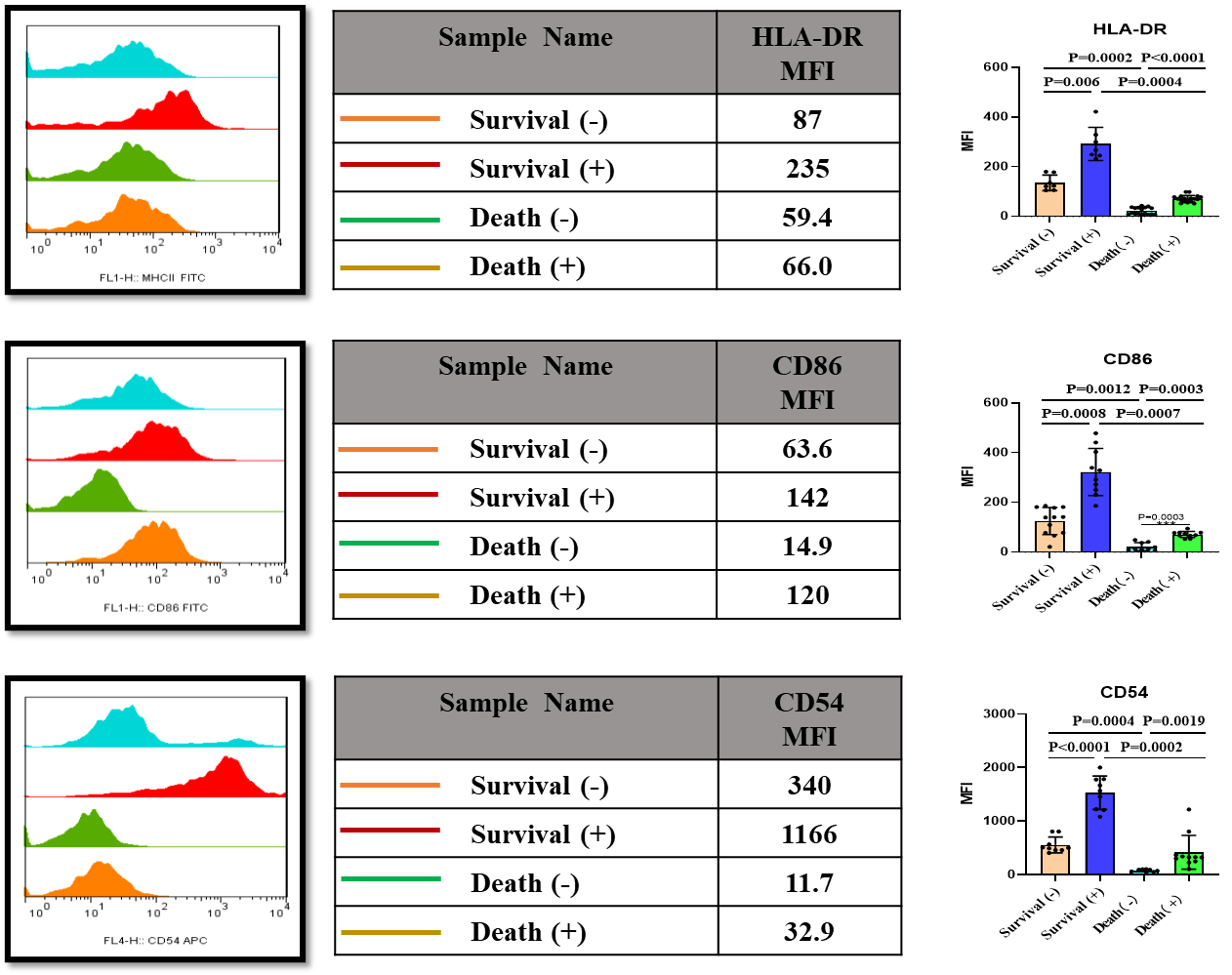


**Figure s7. Representative data depicting the surface expression of HLA-DR, CD86, and CD54 on monocyte-derived dendritic cells (moDCs) from patients with acute-on-chronic liver failure (ACLF), with or without stimulation.** Grouping was performed based on Outcome. (+): Stimulated with alcohol. Representative data was determined as mean fluorescence intensity (MFI). Abbreviations: moDCs: Monocyte-derived dendritic cells; ACLF: Acute-on-Chronic Liver Failure; HLA-DR: human leukocyte antigen-DR; PCT: Procalcitonin; SD: Standard Deviation . Data analyzed by two-way analysis of variance (ANOVA), displayed as Mean with SD. *P <0.05, **P <0.01, ***P <0.001 and ****P <0.0001.


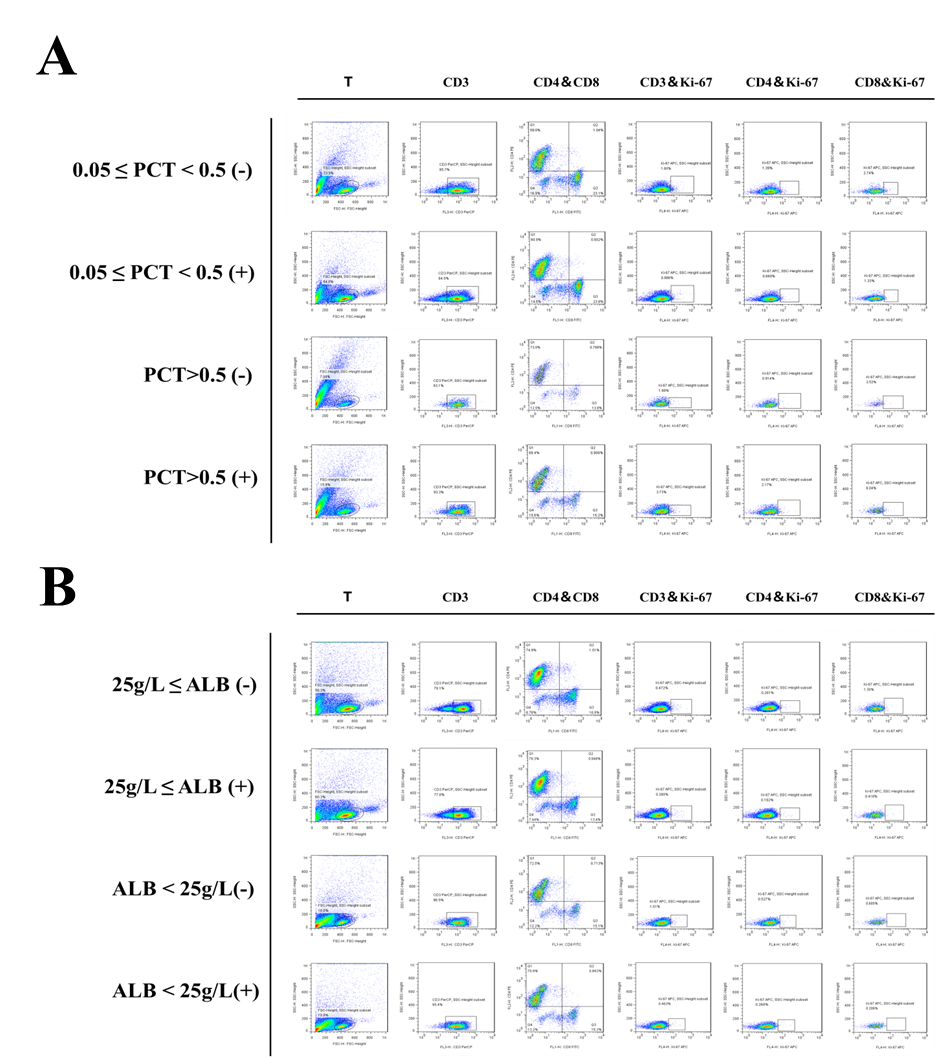


**Figure s8. Subsets of peripheral blood T cells and their proliferation after co-culture with moDCs from ACLF with or without stimulation.** Representative dot plots depicting the grouping based on infection (A) and albumin levels (B). (+): Stimulated with alcohol. Abbreviations: moDCs: Monocyte-derived dendritic cells; ACLF: Acute-on-Chronic Liver Failure; T: T lymphocytes; PCT: Procalcitonin; SD: Standard Deviation. Data analyzed by two-way analysis of variance (ANOVA), displayed as Mean with SD.


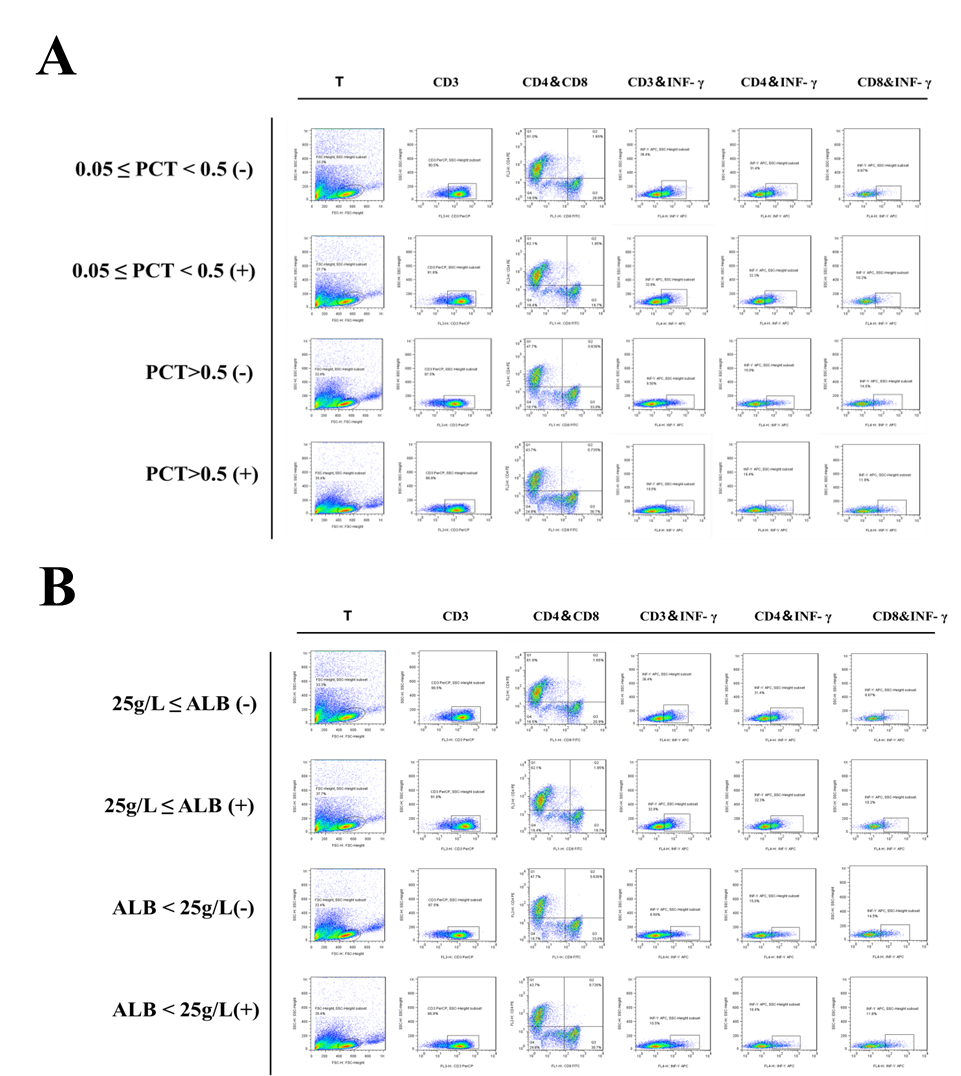


**Figure s9. Subsets of peripheral blood T cells and their interferon-γ production after co-culture with mo-DCs from ACLF with or without stimulation.** Representative dot plots depicting the grouping based on infection (A) and albumin levels (B). (+): Stimulated with alcohol. Abbreviations: mo-DCs: Monocyte-derived dendritic cells; ACLF: Acute-on-Chronic Liver Failure; T: T lymphocytes; PCT: Procalcitonin; SD: Standard Deviation. Data analyzed by two-way analysis of variance (ANOVA), displayed as Mean with SD.
